# Can Rehabilitation Influence the Efficiency of Control Signals in Complex Motion Strategies?

**DOI:** 10.1155/2017/3631624

**Published:** 2017-05-24

**Authors:** Joanna Cholewa, Jaroslaw Cholewa, Agnieszka Gorzkowska, Andrzej Malecki, Arkadiusz Stanula

**Affiliations:** ^1^Department of Physiotherapy in Neurological and Musculoskeletal Disorders, J. Kukuczka Academy of Physical Education, Katowice, Poland; ^2^Department of Tourism and Health-Related Physical Activity, J. Kukuczka Academy of Physical Education, Katowice, Poland; ^3^Department of Neurology and Department of Neurorehabilitation, Medical University of Silesia, Katowice, Poland; ^4^Department of Methodology, Statistics and Computer Science, J. Kukuczka Academy of Physical Education, Katowice, Poland

## Abstract

The factor determining quality of life in Parkinson's disease (PD) is the worsening of a patient's walking ability. The use of external stimuli can improve gait when performing complex motor patterns. The aim of this study was to evaluate the effect of rehabilitation on the effectiveness of control signals in people with PD. The study was performed on 42 people with idiopathic PD in the third stage of disease. The control group consisted of 19 patients who did not participate in rehabilitation activities. The experimental group was systematically participating in rehabilitation activities twice a week (60 minutes) for 9 months. Gait speed, mean step length, and step frequency were calculated on the basis of the obtained results. These parameters were compared in both groups by single factor variance analyses. The best results were obtained using rhythmic external auditory signals. The group with patients actively participating in rehabilitation showed statistically significant improvement in gait speed (12.35%), mean step length (18.00%), and frequency step (2.40%) compared to the control group. The presented research showed the positive effect of rehabilitation and was based on the performance of complex motion patterns, using external control signals for their effectiveness in new motion tasks.

## 1. Introduction

One of the factors determining quality of life among patients suffering from Parkinson's disease (PD) is impaired or unusual gait, which changes as the disease progresses and eventually leads to the eventual loss of walking ability [1]. People with PD have reduced walking speed, decreased stride length [1–4], increased variability of gait and increased step frequency [5], difficulty in beginning and ending steps, decreased range of joint motion in lower limbs, lack of motion in upper limbs, and shoulder girdle counter-rotation in relation to hip girdle [3, 4, 6], which results in an increased risk of falls, loss of independence, and significant deterioration in the patient's quality of life.

Impaired gait is best treated by a combination of exercise, along with practical advice and the simultaneous application and development of cognitive strategies [7, 8]. The use of external stimuli (control signals), both audio and visual, can improve gait by focusing attention on the task of walking [9–11]. Counting out loud and conscious control can be used to learn the correct movement sequence. Apart from that, self-generated cognitive instructions, such as thinking about the particular components of motion, for example, heel contact with the ground, long stride, and correct step length visualization, are as effective as visual-spatial external signals (e.g., prompts placed at eye level, signs, and patches placed on the floor) [9]. Through the use of control information signals, both internal and external, which are processed within the cerebral cortex, the impairment of basal ganglia, which usually automatically determine the pace and range of motion, can be avoided [12, 13].

It has been shown that people with PD have difficulty performing two tasks, and they function better when consciously performing individual components of a motor task [3, 6, 14]. It is much more difficult for them to perform various tasks simultaneously; for example, gait is impaired when any object is carried. Galletly and Brauer [4] have shown that people with PD improved stride length by applying visual control signals while performing an additional task, which suggests that rehabilitation of multitask movement patterns may be possible. Similarly, Canning et al. [13] presented a pilot study of people with mild to moderate PD in which they suggest the benefits resulting from applying multitask patterns.

However, the multipurpose nature of active functioning within the home and social environment in which people with PD live can significantly affect their ability to concentrate, which, in turn, may limit the benefits resulting from using personal independent strategies aimed at focusing attention on specific situations in everyday life.

Benefits resulting from applying internal strategies of focusing attention seem to be smaller than those coming from external stimuli, which require from PD patients less effort and concentration. Therefore, application of such stimuli in complex activities may facilitate walking [15].

The aim of this study was to evaluate the influence of rehabilitation on the effectiveness of control signals among people with PD, including gait speed and the average length and frequency of step, in so far as these variables were dependent on given control signals, as well as determine the effectiveness of the rehabilitation programme based on complex motor patterns.

## 2. Patients and Methods

### 2.1. Patients

The study comprised 42 people with idiopathic PD who had been diagnosed as being at the third stage of the disease according to the criteria of the United Kingdom Parkinson's Disease Society Brain Bank and the Hoehn and Yahr scale [16]. No dementia (Mini-Mental State Examination) [17], dyskinesia (Modified Dyskinesia Scale) [18], or periods of sickness were diagnosed, which could have caused problems during testing (Unified Parkinson's Disease Rating Scale) [19] in any of the subjects.

The typical cut-off MMSE (Mini-Mental State Examination) for dementia (≤24 points) was used in our study. Additionally, all MMSE results had values above 25 [20]. No other serious health problems were diagnosed; vision and hearing were not impaired. A Snellen Eye Chart was used to test for vision problems [21]. Hearing was evaluated by asking the patients to detect sound produced at a distance of 20 feet away from the patient in a large room [22].

The patients were divided into the following two groups: experimental (A) and control (B). The experimental group consisted of 23 people (14 men and 9 women) who were systematically participating in rehabilitation activities. All patients gave written consent to participate in the research study. Attendance was checked during each session. Results of 5 patients who resigned from participation in research and 3 others who either missed 4 consecutive exercises or missed 6 or more meetings during the whole experiment were excluded from analyses.

The activities were performed by an experienced therapist who was also one of the authors of this research. The applied rehabilitation programme was determined by the authors of the research and was customized to the degree of disease stage and changing functional abilities of patients.

The control group (B) consisted of 19 patients (11 men and 8 women) who never participated in rehabilitation activities. Lack of participation in these activities was confirmed on the basis of interviews carried out on the first days of each consecutive month. The data are presented in Table 1.

Group A patients performed rehabilitation activities in the gym twice a week (60 minutes) for a period of 9 months before the study commenced. Each exercise was functionally justified and designed to help patients cope with daily activities. Rehabilitation was targeted at particular symptoms so that stored patterns for acquired and automatic movements were used to the optimum. The procedure included the following: frequent repeating of movements, combining movements with acoustic movement initiators (stepping), repeating movements at different frequencies, introducing free movements with stimulating mechanisms such as control signals (audio, visual, and/or sensory), using imaginative stimulation of movement before its execution, evoking equivalent reflexes, and correcting abnormalities in posture.

The rehabilitation therapy of gait included the following: focusing on the length of steps, foot to base distance, broadening gait posture, changing of direction, and successively focusing on the twist of the head, shoulder girdle, and hip girdle in a fixed movement direction. Visual control signals (tape and boxes arranged on the floor) and audio control signals (clapping) were applied. The exercises also included sensory control signals, which initiated movement and walking exercises using other motor programmes (sticks, sand bags, and balloons). Exercises were based on complex motor patterns and were functionally justified and aimed at developing abilities to cope with daily activities.

Posture corrective exercises were additionally applied through the use of cognitive strategies involving conscious focusing and maintaining an upright position of the body, mirrors, and the “high walk” strategy incorporating elements of stretching as well as exercises reducing increased muscle tonus and strategies for coping with trembling. Used sets of exercises were worked out by authors of the research work on the basis of literature [23], and more information is available on request.

Subjects from Group B did not take part in rehabilitation exercises or any other form of movement therapy.

The research was accepted by Ethical Committee at The Jerzy Kukuczka University of Physical Education in Katowice.

### 2.2. Tasks

Functional tasks, carried out at the gym, consisted in walking along a straight section of 10 metres and unfolding an umbrella. The subjects held a standard, automatic, 42-cm long umbrella with a curved handle in their hand, which they had to open during the walk without stopping and hold it overhead until they reached the finish line. The following tests were performed:

  Trial I (T1): subjects were tasked to walk a 10-m section while simultaneously opening an umbrella; subjects could walk at their preferred pace while focusing on both tasks.
  Trial II (T2): subjects were tasked to walk a 10-m section while simultaneously opening an umbrella; while walking, subjects were asked to synchronize their steps to tone sounds.
  Trial III (T3): subjects were tasked to walk a 10-m section while simultaneously opening an umbrella; while walking, subjects were asked to synchronize their steps to flashes of light.

Tests were carried out before and after the 9-month rehabilitation exercises. Trials were performed at random. In order to avoid muscle fatigue, 10-minute intervals were implemented. Audio and visual signals were randomly attributed to the first or second pair of signals. In conditions of giving signals, the patients were asked to synchronize gait with each audio signal or light flash. The frequency of audio and visual signals was determined by measuring the time needed to cover 10 steps by a subject, at a preferred walking pace. Each subject began the test from the starting line, simultaneously with the given signal. The measurement was finished after reaching the 10-metre line. In order to avoid fatigue, 10-minute intervals between consecutive measurements were applied. Due to short time of efforts and little intensity, fatigue did not influence obtained results [24].

Measurements were performed two days after the last rehabilitation activities. To make sure that the subjects were under the influence of medicine at an “on” phase, the measurements were performed one hour after taking medicine. The standard assessments of PD patients in most trails were performed in ON phase. The aim of PD patients treatment was to obtain ON phase all day long as the most interesting aspect was functional status in PD patients in ON state. We considered OFF phases medication irrelevant because in that particular treatment OFF phase did not take place.

The signals were provided by a device, worn at waist level, that emitted audio or visual signals. Acoustic signals were delivered via headphones, while the visual signal was delivered via a diode attached to the optical eyeglasses of the examined subject or to an alternative pair of glasses.

Measurements of time and the number of steps were registered by an accelerometer, placed at the patient's waist.

The subjects had not been informed in advance of the purpose of the test.

## 3. Results

To achieve the aims of the research, the gait pace, frequency of steps, and average step length of every subject in each test were calculated by an accelerometer. The tested parameters were compared in groups, before and after the experiment, using an analysis of variance with the Statistica computer programme. Tukey's post hoc tests were applied to determine the interaction between results obtained from the completed tests. The analysis took the results obtained in the second trial of each test into account, regarding the first attempt as a practice run. During the tests, the subjects did not report any problems with their execution. Subjects did not perform trials of these tests during their rehabilitation activities.

To compare the data of subjects shown in Table 1, the *t* test for significant differences of the mean values was applied. The conducted analysis showed no statistically significant differences in the presented parameters.

The results obtained on examined gait parameters during the trials are shown in Table 2.

### 3.1. Gait Speed

The comparison of results in the experimental group, both before and after rehabilitation processes, showed statistically significant differences in each test carried out. The results in the control group were not statistically significant (Table 2).

**Figure 1:**
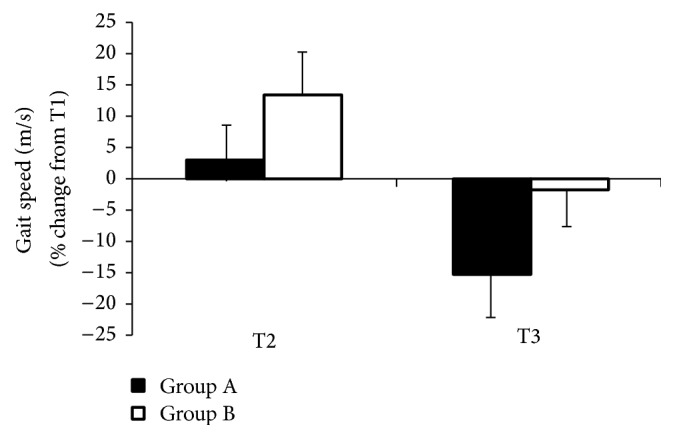
The effects of external rhythmic signals, auditory (T2) and visual (T3), in people with PD and in control group during walking with dual-motor task, walking speed, are shown.

In group A, the analysis of variance revealed a statistically significant difference in gait pace in the given tests among the subjects participating in rehabilitation activities. The highest pace was observed in a T2 test with auditory signals, but, in relation to the test without control signals (T1), these differences were not statistically significant (*F* = 9.65  *p* < 0.004) and amounted to 2.53%. Statistically significant differences were observed between the test using auditory signals and the test using visual control signals (*F* = 5.76, *p* < 0.001), which was 20.90%, as well as between the tests with visual signals and without visual signals 17.91% (*F* = 6.92, *p* < 0.001; Figure 1).

Statistically significant differences were also found in Group B among subjects not participating in the process of rehabilitation. Similar to group A, the fastest gait was recorded in the test with auditory signals, but, in relation to the test without audio control signals, the difference was 14.08%, which was statistically significant (*F* = 2.94, *p* < 0.001). There was also a statistically significant difference between tests with control signals and visual signals (*F* = 2.69, *p* < 0.001).

In all the tests performed after the experiment, subjects in group B moved much slower than those in Group A. In the T1 test, subjects had an average speed of 0.18 m/s faster than those who were not exercising, and the difference was 22.78%. In tests of auditory signals, the difference was 12.35%, and the lowest difference occurred during the use of visual signals and amounted to 10.45%.

### 3.2. Average Step Length

The period of 9 months of rehabilitation processes influenced gait lengthening in all dual tasks (T1, T2, and T3) in group A. However, in group B, a gait shortening was observed, but it was not statistically significant (Table 2).

**Figure 2:**
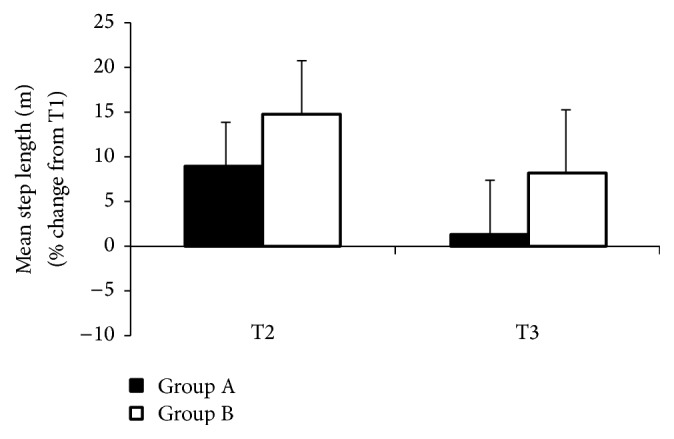
The effects of external rhythmic signals, auditory (T2) and visual (T3), in people with PD and in control group during walking with dual-motor task, mean step length, are shown.

The average step length in group A differed depending on the attempts performed. In test T1, it was 0.43 m, which was 16.28% lower compared to the test that employed auditory signals, and this difference was statistically significant (*F* = 7.21, *p* < 0.03). Visual signals led to an increase in the average step length of 0.01 m. However, the differences were not statistically significant (Figure 2).

In Group B, the subjects achieved a changed step length in consecutive tests, but the difference was only statistically significant between T1 and T2 tests (*F* = 2.64, *p* < 0.002) and amounted to 21.95% (0,09 m). No significant difference between the types of signals was observed.

Analysing the results obtained from both participating subjects (group A) and nonparticipating subjects (group B) in rehabilitation activities, a longer average step length was observed in Group A. The greatest difference occurred in the test without control signals, and amounted to 25.58%. In both groups, auditory and visual signals resulted in an increased step length, but a greater difference was noticed when using auditory signals.

### 3.3. Frequency of Steps

Analyses of results in group A, performed before and after the rehabilitation period, showed statistically significant differences in test T1. In all other tests, the results were not statistically significant. In the control group, the differences before and after the experiment were not statistically significant as well (Table 2).

**Figure 3:**
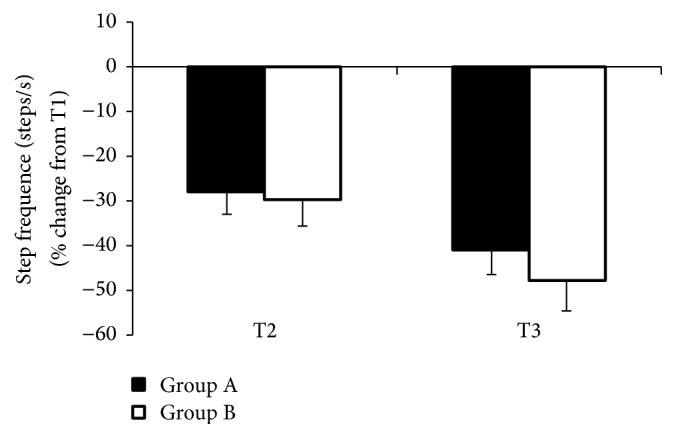
The effects of external rhythmic signals, auditory (T2) and visual (T3), in people with PD and in control group during walking with dual-motor task, step frequency, are shown.

A complex motor scheme, performed in Groups A and B with the use of control signals, showed no difference between T2 and T3 tests in either group. A statistically significant difference was observed in the test without visual signals and auditory signals (*F* = 3.45, *p* < 0.033), as well as with visual signals (*F* = 6.04, *p* < 0.005) in group A, and, respectively, for auditory signals (*F* = 2.94, *p* < 0.003) and visual signals (*F* = 3.45, *p* < 0.002) in Group B (Figure 3).

## 4. Discussion

### 4.1. Controlling Signals

The most important aim of the study was to evaluate the effect of PD patient's rehabilitation process on efficiency of control signals, generated from outside. However, in order to assess the importance of used rehabilitation program, evaluation of applied control signals on measured gait parameters was presented in the first part of discussion.

The effects of gait control signals have been presented in the literature [10, 11, 25]. The use of visual and auditory signals influences the length and speed of gait [9–11, 25]. Numerous studies [10, 11, 25] have demonstrated that rhythmic signals, supplied at frequencies above those preferred by the subject, resulted in an increased gait speed, increased average step length, and stepping frequency. In these studies, PD patients performed single motor activities. Performing dual motion tasks had a negative effect on gait in people with PD [3, 6]. Iansek et al. [26] believed that this effect is due to improper functioning of the basal ganglia, which requires more attention and the use of cognitive resources to perform dual motion tasks, that is, both gait and parallel tasks.

The results obtained demonstrated the positive effect of control signals when trying to perform complex motion activities in both Groups A and B. Gait speed increased, with the optimum effect achieved through the application of auditory signals. Similar results have been obtained by Howe et al. [27] indicating that auditory signals may temporarily influence the improvement of gait speed and consequently provide a potential strategy for increasing gait efficiency. Morris et al. [9] have found that auditory control signals result in restrictions when increased gait pace is caused by an increased average length of step. The results obtained in the study showed that subjects under the influence of auditory stimuli increased both their average step length and step frequency.

Control signals can work as a temporary replacement for defective signals, which are generated internally by the basal ganglia. Kritikos et al. [28] believe that not only is the frequency of the transmitted auditory signals important but also the moment in which the signal is sent to a motion sequence. Signals sent later facilitate better performance of the task than those sent earlier [28].

During the study, people with PD showed a higher level of reaction to auditory signals. The results confirmed the hypothesis that the use of external control signals during the functional test decreases gait disturbances due to a lower demand for concentration, compared to performing tasks without signals, which requires the use of cognitive processes to effectively split attention between the actions to be taken [14].

On the basis of the results obtained, it was noted that the visual stimuli contributed to the trend of improving the efficiency of gait in complex motion tasks to a lesser extent. The beneficial effects of a visual signal disappear while performing additional tasks [9]. People with PD may use the information that is provided by sending visual stimuli and compare them with internally generated strategies that require concentration, planning, and motion control. Movement is probably the result of cognitive feedback [14, 29].

The use of auditory and visual signals was studied among people with PD in relation to gait and while performing a task that requires two physical motor skill activities within a home environment. Rochester et al. [14] evaluated walking along a section with a tray of cups. Applied auditory signals contributed to a significant increase in the average step length during tasks requiring the execution of two motor functions, while visual signals contributed to the improvement of gait speed during a complex task.

### 4.2. Use of Control Signals

Motion rehabilitation is believed to have a positive influence on physical abilities, functional independence, and the quality of life among people with PD. With regard to the rehabilitation of people with PD, the following two strategies are available: symptom-based therapy and task-based therapy. According to current opinion, one form of physiotherapy in relation to PD is the implementation of the rehabilitation based on the performance of functional tasks through control signals. Many authors have claimed that external control signals during rehabilitation processes seem to be an effective method of improvement [30, 31].

Results of available research works show positive effect of motion rehabilitation, which decreases neurological symptoms and improves life quality, mood, and performance functions as well as self-reliance in performing everyday tasks. Regular physical activities contribute to an increase of neurotransmitters' concentration (serotonin, dopamine, acetylcholine, and noradrenalin) and influences changes in activity of some receptor subtypes in neurotransmitters, which in turn leads to changes in activity of whole cortical and subcortical structures [32]. Increased severity of the disease, gait and coordination disturbances, and less self-reliance in performing daily activities correlate with decreased physical activity and may enhance disease development [33]. Dynamic support of visual and auditory signals combined with rehabilitation is a promising strategy in improving life of PD patients, even in case of severe disease stage [34].

McIntosh et al. [25] recorded improved gait speed and step length after a 3-week experiment during which PD patients performed exercises with auditory signals for 30 minutes a day. Similar results have been obtained by Thaut et al. [10]. The conclusion to be drawn is the importance of auditory signals in improving the quality of gait in the rehabilitation process. Del Olmo and Cudeiro [35] also examined the effect of auditory stimuli on gait parameters but additionally used a variety of training conditions and introduced additional tasks. The training programme lasted for a period of 4 weeks, during which subjects practised one hour a day, 5 times a week. The programme was helpful in improving gait parameters, which proved that changes in motion patterns due to disease progression are temporarily reversible.

The influence of dynamic and static visual control signals on gait parameters has been analysed by Azulay et al. [36] and the obtained results showed that, during PD rehabilitation, visual signals may help movement and are rather related to the visual perception of motion than to position or orientation. The dynamic visual effects may occur subconsciously, perhaps using mechanisms similar to those that control the velocity of eye movements.

Using a rehabilitation programme based on three types of control signals for a period of 3 weeks, 3 times a week, for 30 minutes in home conditions, Nieuwboer et al. [37] recorded improved gait speed, step length, and episodic reduction of freezing of gait. At the same time, they found that home-based physiotherapy delivers limited and specific benefits.

In performing an experiment involving simultaneous use of dual control signals (auditory and visual) during a period of 4 weeks for 20 minutes a day, Frazzitta et al. [38] recorded an improvement in measured gait parameters, which indicated the positive effect of both signals on motor performance.

Many reports on the use of signals in the rehabilitation of people with PD have confirmed their positive effect on improvement. However, such studies are mostly based on the performance of simple motion patterns [39].

The examinations presented in this research used control signals during a visual and auditory functional complex motion scheme, which was preceded by the 9-month rehabilitation programme. Therefore, it is impossible to compare the results obtained during performance of dual-motor activities with other results because this type of research has not as yet been conducted.

The fact that those patients who participated in rehabilitation demonstrated greater improvement in gait parameters than those who did not participate in rehabilitation activities indicates its important role in the improvement process. It cannot be excluded that the training of complex motion patterns during the 9-month period of rehabilitation prior to the study may have produced that effect. However, it seems that an explanation of better results achieved among subjects participating in rehabilitation cannot be justified by the rehabilitation process only because the tests used during the study had not been known and previously carried out by the subjects. All the subjects had 3 attempts at complex motion tasks, performed for the first time at the gym. The data presented suggest that the rehabilitation of people with PD must not consist only of the use of control signals, but rehabilitation should be based, first of all, on their use in practising complex motion patterns. Moreover, it is recommended that this form of exercise should be applied in the early stages of disease, when the compensatory movement strategies are not yet needed. Basing rehabilitation exclusively on simple motion patterns may have a limited impact.

## 5. Conclusions

The effectiveness of using external control signals in new motion tasks is increased after implementing a rehabilitation program, based on the performance of complex motion patterns. The use of rhythmic auditory signals was found to cause increased gait speed, increased average step length of steps, and increased step frequency among people with PD. The results confirmed the usefulness of external control signals during complex functional tasks and extend the possibilities of their application in rehabilitation programmes.

## Figures and Tables

**Table 1:** Characteristics of the subjects.

Analysed parameters	Group A	Group B	*t*-statistic	
			*p*	*t*
Gender (men/women)	14/9	11/8		
Age (years)	61.32 ± 4.02	62.68 ± 3.02	0.242	1.188
Body height (cm)	165 ± 5.98	167 ± 9,63	0.318	1.006
Body weight (kg)	68.48 ± 7.64	70.75 ± 8,38	0.251	1.158
Duration of illness (years)	7.57 ± 1.25	8.29 ± 0.983	0.100	2.657
Hoehn and Yahr score	3.2 ± 0.32	3.1 ± 0.36	0.234	1.200

**Table 2:** Comparison of tests' results prior to and after the rehabilitation therapy in the experimental and control group.

Variable		Before		After		Relative difference	Absolute difference	Post hoc test
		$\bar{x}$1	*S*	$\bar{x}$2	*S*	$\bar{x}$1−$\bar{x}$2	$\bar{x}$1−$\bar{x}$2(%)	*p*
Experimental group								

Gait speed (m/s)	T1	0.63	0.03	0.79	0.05	−0.16	25.40	**0.001**
	T2	0.70	0.04	0.81	0.06	−0.11	15.71	**0.001**
	T3	0.59	0.03	0.67	0.05	−0.08	13.56	**0.001**

Step length (m)	T1	0.36	0.04	0.43	0.07	−0.07	19.44	**0.002**
	T2	0.42	0.02	0.50	0.03	−0.08	19.05	**0.002**
	T3	0.40	0.03	0.44	0.03	−0.04	10.00	**0.003**

Frequence (steps/s)	T1	1.92	0.07	1.84	0.07	0.08	−4.17	**0.002**
	T2	1.64	0.06	1.62	0.06	0.02	−1.22	**0.020**
	T3	1.56	0.06	1.52	0.06	0.04	−2.56	**0.001**

Control group								

Gait speed (m/s)	T1	0.64	0.05	0.61	0.05	0.03	−4.69	0.06
	T2	0.72	0.04	0.71	0.03	0.01	−1.39	0.11
	T3	0.62	0.03	0.60	0.04	0.02	−3.23	0.07

Step length (m)	T1	0.34	0.04	0.32	0.04	0.02	−5.88	0.32
	T2	0.43	0.02	0.41	0.03	0.02	−4.65	0.12
	T3	0.38	0.02	0.37	0.03	0.01	−2.63	0.06

Frequence (steps/s)	T1	1.90	0.08	1.91	0.07	−0.01	0.53	0.80
	T2	1.68	0.06	1.63	0.06	0.05	−2.98	0.08
	T3	1.57	0.05	1.59	0.06	−0.02	1.27	0.06

$\bar{x}$1: mean of examined variables in the experimental group.

$\bar{x}$2: mean of examined variables in the control group.

*S*: standard deviation

*p*: probability ratio.
